# Building a City with Low Noise Pollution: Exploring the Mental Health Effect Thresholds of Spatiotemporal Environmental Noise Exposure and Urban Planning Solution

**DOI:** 10.3390/ijerph20054222

**Published:** 2023-02-27

**Authors:** Xue Zhang, Suhong Zhou

**Affiliations:** 1 School of Architecture and Planning, Yunnan University, Yunnan 650500, China; 2 School of Geography and Planning, Sun Yat-Sen University, Guangzhou 510006, China; 3 Guangdong Provincial Engineering Research Center for Public Security and Disaster, Guangzhou 510275, China

**Keywords:** noise exposure, mental health, spatiotemporal behavior, threshold effect

## Abstract

Urban noise pollution and health hazards have become serious social problems and challenges. Noise prevention and control is the most cost-effective health strategy. However, in urban planning and noise control, reliable evidence is still lacking on individual spatiotemporal environmental noise exposure and its mental health effects. This study used real-time noise exposure data and GPS trackers from 142 volunteers aged 18 to 60 years in Guangzhou, and further analyzed the differences in environmental noise exposure and its mental health impact thresholds under individual spatiotemporal behavior. The results showed that the noise exposure of residents under daily activities has obvious differences in time, space and place. Regarding the threshold relationship between noise exposure and mental health, noise exposure at night, work, personal affairs, travel and sleep activities, as well as at home and work had a threshold effect on residents’ mental health. Noise thresholds were 60 dB, 60 dB, and about 34 dB at night, during work or at a workplace, and while sleeping, respectively. The optimal sound environment for personal affairs, traveling, and at home was around 50 dB, 55–70 dB, and 45 dB, respectively. The environmental noise exposure assessment and mental health impact threshold analysis based on the spatial and temporal activities of individuals will provide important reference for government management departments in planning and policy formulation.

## 1. Introduction

Rapid urban social and economic development has triggered serious environmental noise pollution and increased the risk of mental disorders. In 2011, the World Health Organization and the European Union jointly stated that noise has become the second major public health hazard after air pollution [1]. In Europe, more than 30% of the population was exposed to road traffic noise of over 55 decibels at night, and lead to serious sleep disturbances and adverse health effects [2]. In China, the report noted that in 2020, urban departments of ecology and environment, public security, housing and urban and rural development received over 2 million complaints about environmental noise, involving domestic noise, construction noise, industrial noise and traffic noise [3]. Noise generated in urban construction and daily life has seriously affected human behavior, well-being, productivity and health [4]. Excessive exposure to noise pollution not only made people irritable and worsen sleep, but also increased the prevalence of hypertension and cardiovascular and cerebrovascular diseases, endangering people’s physical and mental health [5,6,7,8,9].

The effect of environmental noise on human mental health became a major concern in terms of geography and public health, with the increasing prominence of mental health disorders and becoming one of the biggest challenges in public health problems. Studies have shown a dose–response relationship between noise exposure and mental health in residents [10,11]. Large amounts of noise studies derived from road traffic and airports have shown that the risk of poor mental health increases with increased exposure to traffic noise [7,12,13,14,15,16].

Residents’ health is closely related to their daily life. Environmental exposure and residents’ mental health from the perspective of individual activity and movement has become a new direction to examine [17,18,19]. Since 1996, some scholars have carried out relevant research on individual noise exposure surveys. In the process of investigation, the questionnaire was used to understand the time arrangement of main activities, personal living, working environment and basic information of individuals. The individual’s exposure to noise was also collected throughout the day and night [20]. In recent years, with the development of GPS and GIS technology and the application of portable environmental monitoring equipment, more and more scholars have realized the limitations of static geographic noise exposure assessment based on an individuals’ place of residence or workplace [21,22,23]. Real-time noise monitoring and assessment methods that consider individual daily activities and dynamic changes in the time and location of noise can more accurately capture the real noise environment of residents [9,24,25,26]. However, studies on the relationship between residents’ mental health and spatiotemporal environmental noise exposure are still very scarce.

In the face of severe environmental noise and health problems in cities, noise control is the most economical and effective health strategy. Determining accurate environmental health impact thresholds is essential for urban policy makers and residents to understand environmental health risks and respond to environmental hazards [27]. In the study of the relationship between environmental noise and mental health, the essence of the noise threshold is that a certain limit of noise pollution does little harm to the health of residents. However, when the environmental noise pollution or noise exposure exceeds a certain limited value, it will greatly aggravate the health risks and cause serious harm to the residents’ health. The World Health Organization reported that noise over 70 dB can cause severe hearing damage [28]. In the threshold approach of noise response problems, the usual criterion used is to define the points where 10% of the population is severely or severely affected by noise as excessive noise [29]. However, there is no clear threshold level of noise present for resident health effects, due to the effects of many characteristics of the noise itself, environmental factors, and extensive differences in individual sensitivity to noise. Therefore, it is particularly critical to study the nonlinear relationship between noise and mental health research through machine learning methods to explore the threshold problem of the effect of noise on mental health [24,30,31].

The purpose of this study is to investigate the effect threshold of residents’ noise exposure and mental health in different time periods, places and activities. It particularly focuses on (1) spatiotemporal activity-travel characteristics of resident and noise exposure differences; (2) threshold for the effect of environmental noise exposure on mental health under different time periods, places, and activities. The contribution is that it truly reflects the characteristics of residents’ noise exposure in their daily activities, and for the first time proposes and answers the threshold of the effect of noise on mental health under different time, place and activity background. The findings of this study will help urban planners to develop more precise environmental noise control standards for specific space and provide reference for individual active noise prevention and control.

## 2. Materials and Methods

### 2.1. Study Area and Survey

The data used in this study come from a survey on residents’ daily activity and environmental exposure in Guangzhou from November 2018 to January 2019. The survey covers 7 communities in Tangxia Sub-district, Tianhe District, and Guangzhou, with a total of around 2.2 km^2^. Tangxia Sub-district is a representative large-scale comprehensive community integrating commercial housing, affordable housing, public rental housing and rental housing of “urban village”. Using Tangxia Sub-district as a case study, we can examine the differences in activity–travel patterns and noise exposure of residents from various socioeconomic backgrounds, and further explore the relationship between noise exposure and residents’ mental health at different time periods, activities and places.

All volunteers were asked to carry a portable noise sensor (SLM-25 Sound Level Meters) for 48 h covering a workday and a weekend day (from 3 a.m. on Sunday to 3 a.m. on Tuesday) to record real-time noise exposure data, as well as to complete the daily activity–travel and environmental health questionnaire. A total of 156 volunteers participated in the survey, among which 10 people participated in the preliminary survey, and 4 people experienced serious data loss due to equipment failure or personal reasons during the survey stage. Finally, there were 142 volunteers with complete data sets. The specific survey procedures and equipment information used were introduced in detail in Zhang’s research [24].

The data mainly used in this research include two parts: daily activity-travel survey and individual noise exposure assessment. Among them, the volunteers’ basic information and family information, including gender, age, education, income, housing, time spent in green space and neighborhood, were collected using the residents’ daily activity survey, the results of which are detailed in Table 1. In addition, the volunteers were asked to recall information on all travel and activities of the survey day, and record in detail the types and starting time of activities, the types of activity location, travel mode, starting and ending points of travel, and starting time of travel throughout the day. All travel and activities should be recorded in temporal continuity. In this study, the main activity places of residents were divided into home or community, workplace, school, shopping malls or supermarkets, restaurants, parks, hospitals or clinics, relatives’ homes, sports fields and 10 other types. The main activity types of residents were divided into work or business, personal affairs, family affairs, shopping, entertainment and leisure, social activities and 7 other types.

Self-reported mental health data were also obtained from the questionnaires. The World Health Organization’s Five Well-Being Index (WHO-5) detailing feeling “cheerful and in good spirits”, “calm and relaxed”, “active and vigorous”, “fresh and rested”, and “daily life has been filled with things that interest me” during the past two weeks was used to assess the subjective mental health [32,33,34]. The responses were quantified on a 5-point Likert scale ranging from 1 (none of the time) to 5 (all of the time). The total scores of the WHO-5 range from 5 to 25, and the higher the score, the better the psychological condition.

The GPS-equipped mobile phones and the activity–travel diaries were used to record the trajectory of residents’ daily behavior. Through the GPS-equipped mobile phones, it was possible to record the space–time trajectory of residents in real time and reflect the real activity paths and locations of residents. The activity–travel trajectory data points obtained by the two survey methods can be verified and integrated with each other, which further ensures the reliability of the data. The data of the questionnaire, real-time noise levels, activity travel diary, and GPS track points were integrated based on participants’ unique identifiers.

### 2.2. Personal Noise Exposure Assessment

The real-time minute-by-minute noise data recorded by the portable noise sensors were further calculated as the A-weighted equivalent sound pressure level of residents at different times, activities and places. The A-weighted equivalent sound pressure level is a general method adopted by International Organization for Standardization (ISO) to measure the noise exposure of an individual [35]. It refers to the average value of the A sound level according to energy for a certain period of time. Personal noise exposure measured by the A-weighted equivalent sound pressure level (*L_Aeq,T_*) over *T* hour was calculated according to the following Formula (1):

(1) $$L_{Aeq,T}=10lg\left(\frac{1}{T}\sum_{n=1}^{T}10^{0.1L_{eq,T_{n}}}\right)dB\left(A\right)$$

where *T* represents cumulative time *T* minutes; *L_Aeq,T_* is the A-weighted equivalent sound level over a total of *T* minutes; *L_eq,Tn_* is the A-weighted equivalent sound level at the *n* minute, which is the reading of the every-minute sound level collected by the portable noise sensors.

The A-weighted equivalent sound level within 48 h on a workday and a weekend day (*L_eaq,48h_*), A-weighted equivalent sound level within 24 h on a workday (*L_eaq,W_*) and on a weekend day (*L_eaq,R_*), A-weighted equivalent sound level between 6:00 and 22:00 on a workday and a weekend day (*L_eaq,D_*) and A-weighted equivalent sound level between 22:00 and 6:00 on a workday and a weekend day (*L_eaq,N_*) were calculated. In addition, the A-weighted equivalent sound levels of residents in different activities and places during the two days of the survey were calculated, which are detailed in Table 1.

### 2.3. Model and Methodology

The random forest method was applied to disentangle the complex relationships between the noise exposure and mental health under different spatio-temporal and activity dimensions. The random forest method is an ensemble learning method that explores finer associations between results and explanatory variables, rather than assuming a priori a particular relationship that fits the data [36]. It works by combining decision trees from multiple individuals to optimize model fitting and prediction, and finally make the loss function reach a minimum value or maintain stability. The random forest method has been widely used in environmental health research to study the non-linear threshold relationship [24,25,37,38]. The permutation importance measure introduced by Breiman (2001) can quantify the relative importance of explanatory variables in the prediction results and increase the interpretability of the model [36]. The importance for variable *x_i_* is formulated as:

(2) $$VI_{x_{i}}=\frac{1}{n}\sum_{t}\left(Q_{MSE}^{t}-Q_{MSE,p_{i}}^{t}\right)$$

where $VI_{x_{i}}$ is the variable importance of variable *x_i_*; *n* is the total number of decision trees in the forest; $Q_{MSE}^{t}$ represents the mean square error before the tree *t* arrangement; $Q_{MSE,p_{i}}^{t}$ represents the mean squared error after the arrangement of variable *x_i_*.

In addition, the partial dependence graph generated by random forests enables the visualization of the relationship between the results and the explanatory variables [39]. In this study, it describes the marginal effect of noise exposure on mental health, while controlling for the average effect of all other explanatory variables in a given model. The partial dependence of $\hat{f_{s}}\left(x_{s}\right)$ on $x_{s}$ is formulated as follows:

(3) $$\hat{\;f_{s}}\left(x_{s}\right)=\frac{1}{N}\sum_{i=1}^{N}\hat{f}\left(x_{s},\;x_{ic}\right)$$

where $x_{1c}$*,* $x_{2c}$, …, $x_{ic}$ are the values of the other variables $x_{c}$ in the dataset, and *N* is the number of instances.

## 3. Results

### 3.1. Trajectories of Residents’ Daily Activities

**Figure 1:**
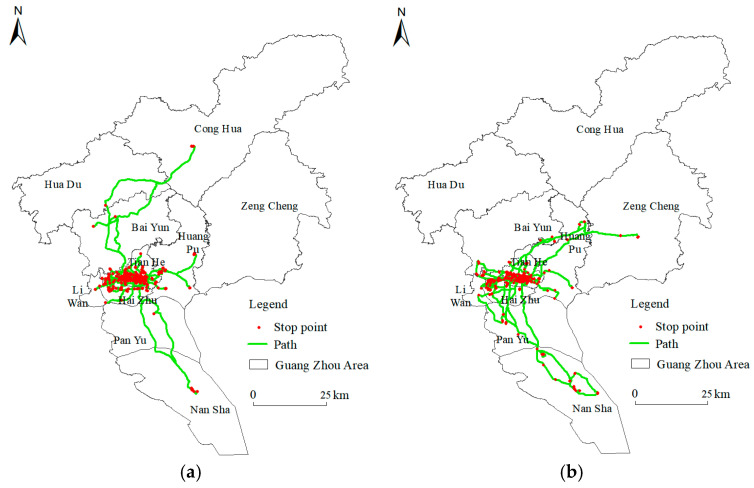
Trajectories of residents’ daily activities. (**a**) Workday; (**b**) weekend.

A total of 142 residents’ activity–travel records were obtained during a workday and a weekend day. There were 2189 trips and activities during workdays, with an average of 15.42 per person. A total of 2050 trips and activities were recorded on weekend days, with an average of 14.44 per person. Using the ArcGIS tool, the real spatial and temporal trajectory of residents on workdays and weekend days were depicted, respectively, as shown in Figure 1. The range of residents’ daily activity was within the main urban area. On workdays, the trajectory of residents’ activities were similar, with the main activity sites in Yuexiu District, Tianhe District and Haizhu District, whereas only some residents’ activity tracks extend to Huangpu District and Liwan District. On weekend days, residents’ places of activity were scattered, mainly in Yuexiu and Tianhe, followed by Liwan District, Haizhu District, Huangpu District, Panyu District and Nansha District. The activity trajectories of residents on workdays and weekends indicated that residents have been involved in different activity spaces in their daily life. The environmental noise exposure assessment based on fixed places of residence is biased from the real environment. Therefore, it is necessary to pay attention to the noise exposure and mental health effects of residents from the perspective of dynamic activities.

### 3.2. Temporal and Spatial Distribution Characteristics of Residents’ Daily Activities

**Figure 2:**
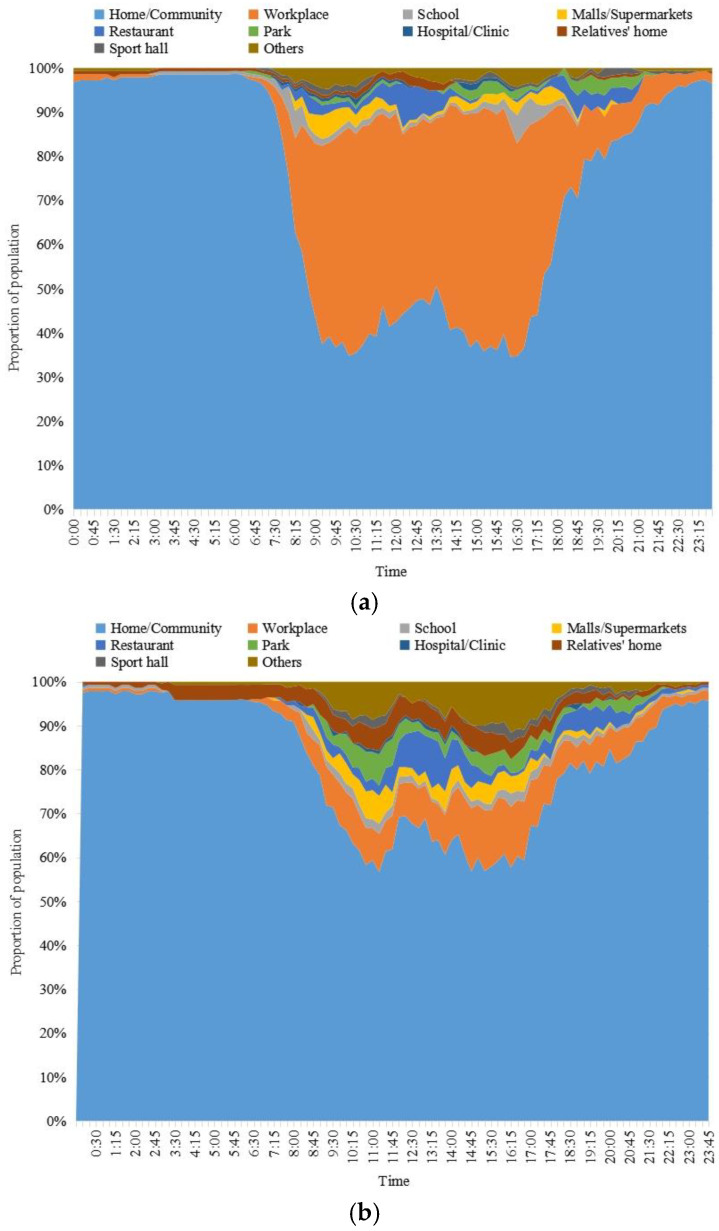
The relationship between activity location and time of workdays and weekends. (**a**) Workday; (**b**) weekend.

Figure 2 showed the distribution characteristics of residents’ activity place and time on workday and weekend. On workdays, the main place of residents’ activities was home (community), and the average time spent at home (community) accounts for 72% of the total time, about 17.3 h, followed by work place (20.9% of the total time, about 5 h), restaurant (1.5% of the total time, about 0.36 h), school (1.1% of the total time, about 0.26 h) and other (1.5% of the total time, about 0.36 h) (Figure 2a). From the change in residents’ activity locations on workdays, the peak hours for arriving and leaving the workplace were 7:00–9:00 am and 17:00–19:00 pm, respectively. Between 11:00 and 14:00, the number of people in offices decreased while the number of people in restaurants and restaurants increased. In addition, the main time for residents to appear and leave in the school was 7:30 am to 8:30 am and 16:00 pm to 17:30 pm, which corresponds to the time for students to travel to and from school.

On weekends, the average time of residents at home (community) accounted for 80.9% of the total time, about 19.4 h, followed by workplace (5.3% of the total time, about 1.27 h), family and friends’ homes (2.9% of the total time, about 0.7 h), restaurants (2.3% of the total time, about 0.55 h) and others (3.3% of the total time, about 0.79 h). The time when residents are not at home and in the community on weekends was mainly between 8:00 am and 18:30 pm, and it decreased significantly after 18:30 pm. Compared with workdays, residents spend continuous amounts of time in malls or supermarkets, parks and relatives’ homes on weekends, which reflected that residents have more lasting and flexible time in leisure and entertainment places on weekends.

The relationship between activity type and time spent of residents on workdays and weekends was further analyzed (Table 2). On workdays, the average time spent on personal affairs was 12.3 h, accounting for 51.2% of the total time, followed by work or business (4.5 h, accounting for 18.9%), family affairs (2.5 h, accounting for 10.4%), entertainment and leisure (2.44 h, accounting for 10.2%), and travel (1.6 h, accounting for 6.8%). Compared with workdays, the average working time of residents on weekends decreased by 3 h, whereas the time for personal affairs, family affairs and recreation increased by 0.5 h, 0.9 h and 1.16 h, respectively.

This part of the analysis showed the basic characteristics of residents’ activity spaces, types and time allocation. It also provided a basis for studying noise exposure and mental health effects based on different spaces, activities and time.

### 3.3. Characteristics of Residents Noise Exposure

We analysed personal noise exposure levels from different time, space and activities in people’s daily life. In terms of time, the characteristics of noise exposure on workday and weekend, in the daytime and nighttime, and the variation characteristics of noise exposure during 24 h were analyzed. Spatially, the noise exposure of residents was calculated according to the main activity places of home or community, workplace, school, mall or supermarkets, restaurant, park, hospital or clinic, relatives’ home, playground and other. Finally, the noise exposure levels of residents in nine main types of activities, including work or business, personal affairs, family affairs, shop, recreation, social, sleep, travel and other were analyzed by descriptive statistics.

#### 3.3.1. Temporal Characteristics of Residents Noise Exposure

**Figure 3:**
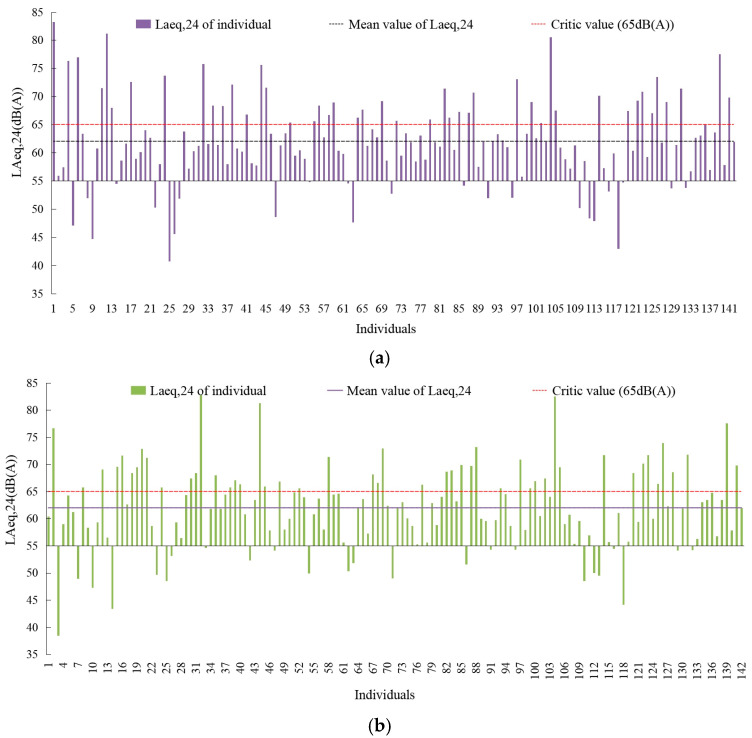
Environmental noise exposure values of residents on workdays and weekends. (**a**) Workday; (**b**) weekend.

Figure 3 illustrated that the average *L_Aeq,24h_* of resident noise exposure on workdays and weekends was 62.03 dB and 61.98 dB, respectively. The proportion of residents with *L_Aeq,24h_* below 55 dB was 16.8% for both days. The proportions of residents with *L_Aeq,24h_* at 55–60 dB, 60–65 dB and over 65 dB were 21.8%, 27.4% and 33.7% on workdays, and 19%, 32.3% and 31.6% on weekends. By comparison, it can be found that there were certain differences in noise exposure levels between residents on workdays and weekends. For 4.2%, 11.3% and 37.3% of the residents, the *L_Aeq,24h_* difference on workdays and weekends was greater than 15 dB, 10 dB, 5 dB, respectively. Only 16.2% of the residents’ differences in noise exposure over two days was less than 1 dB.

**Figure 4:**
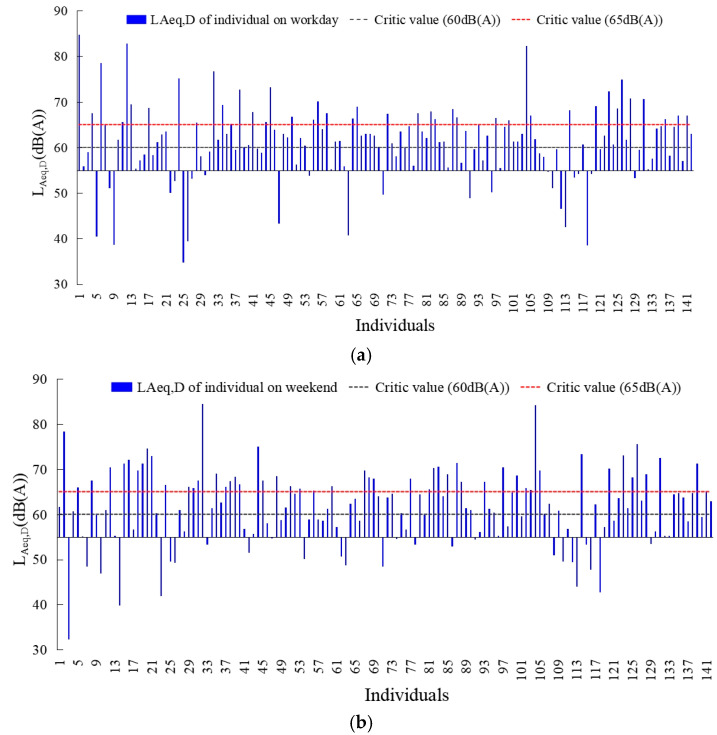
Environmental noise exposure values of residents during daytime on workdays and weekends. (**a**) Workday; (**b**) weekend.

**Figure 5:**
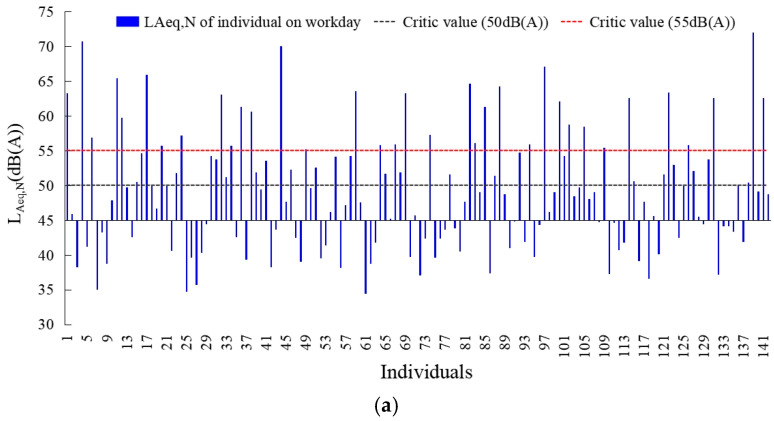

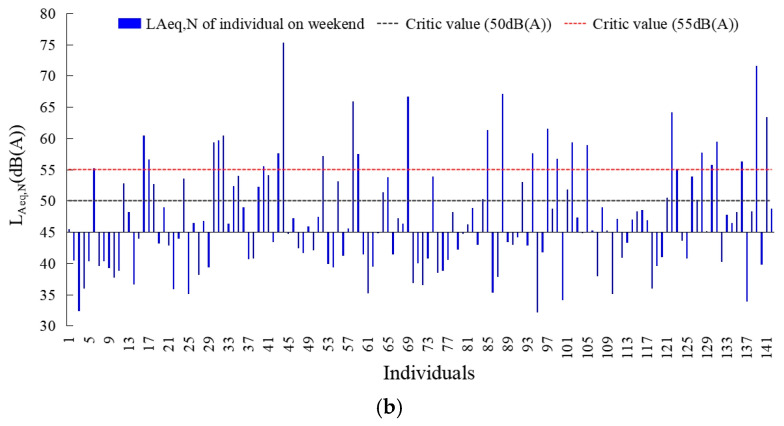
Environmental noise exposure values of residents during nighttime on workdays and weekends. (**a**) Workday; (**b**) weekend.

Figure 4 and Figure 5 showed the equivalent sound level of noise exposure in the day and night on workday and weekend. Residents had high noise exposure in the day which the *L_Aeq,__D_* was 61.19 dB and 61.71 dB on workdays and weekends. Moreover, over eighty and sixty percent of the residents were exposed to *L_Aeq,__D_* exceeding 55 dB and 60 dB. In contrast, seven percent more residents on weekends than on workdays were exposed to a high-noise environment in which the *L_Aeq__,D_* was 65 dB. From the residents’ noise exposure at night, the *L_Aeq,__N_* were 49.28 dB and 47.18 dB on workdays and weekends, respectively. 42.3% and 31.7% of the residents’ average noise exposure value at night exceeded 50 dB, 24.6% and 19.0% of the residents’ average noise exposure value at night exceeded 55 dB. In contrast, the residents’ noise exposure at night on weekends was generally lower than that on workdays.

Clearly, there is substantial variation in noise exposure among individuals. From the average equivalent sound level value of noise exposure in the day and night, residents may be at a higher risk of noise exposure. According to environmental noise guidelines for the European Region, the 24 h equivalent sound level value of recreational noise sources should be within 70 dB, and for road traffic, aircraft and railway noise sources, the equivalent sound level should be lower than 53 dB, 45 dB and 54 dB [38]. Moreover, noise levels starting at 40 dB are mentioned in the WHO night noise guidelines as having adverse health effects. The WHO night noise guidelines (WHO, 2009) mentioned that noise levels at night from 40 dB have adverse health effects [40].

**Figure 6:**
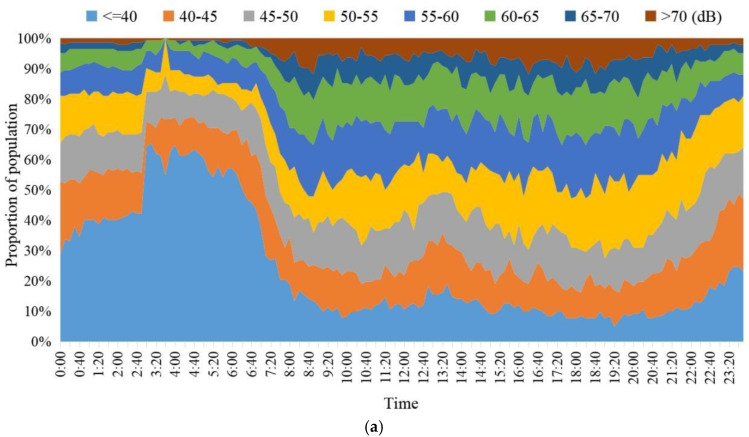

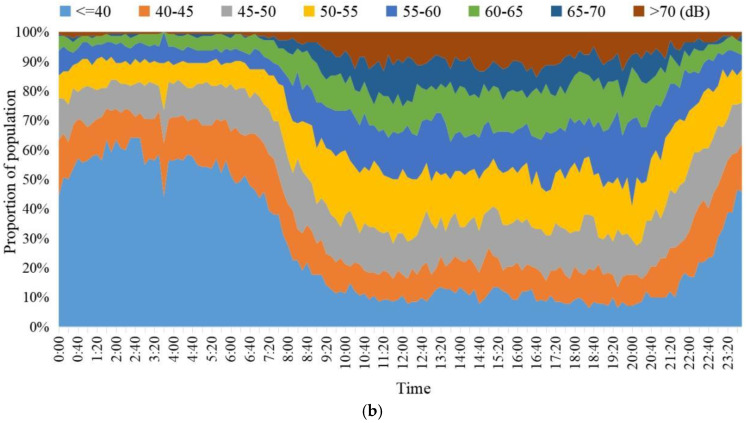
The 24 h noise exposure of 142 residents on workdays and weekends. (**a**) Workday; (**b**) weekend.

Figure 6 showed the equivalent sound level of residents’ noise exposure per ten minutes at various time periods on workday and weekend. It can be found that the proportion of residents exposed to different noise levels on workdays and weekends had the same time rhythm, with two clear stationary periods and two changing periods in time. The period from 0:00 to 7:00 was a stable period in which the residents’ noise exposure on workdays and weekends was at a low level, and 70% of the residents’ noise exposure was less than 45 dB. At 8:00–20:00 on workdays and 9:00–21:00 on weekends, it was another stable period when the residents’ noise exposure was at a high level, with nearly half of the residents’ noise exposure values exceeding 55 dB. The noise exposure level of most residents increased from 7:00 to 8:00 on workdays and from 7:00 to 9:00 on weekends, and decreased from 20:00 to 0:00 on workdays and from 21:00 to 0:00 on weekends. The time rhythm of residents’ noise exposure levels may be directly related to the activity arrangement on workdays and weekends. The noise exposure of different activities and activity spaces needs to be further studied.

#### 3.3.2. Characteristics of Residents’ Noise Exposure under Different Activity Types

**Figure 7:**
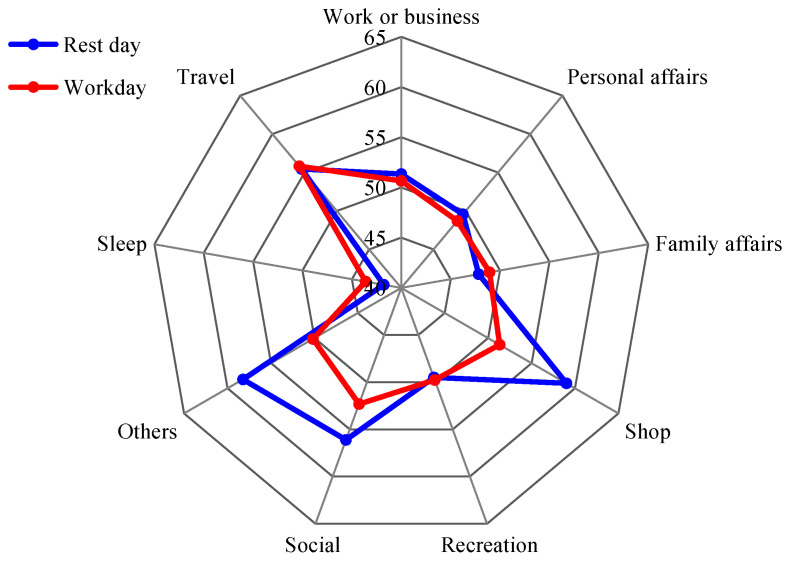
Noise exposure under different activity types on workdays and weekends.

Figure 7 showed the equivalent sound level of residents’ noise exposure under different activity types on workday and weekend. In general, there were great difference of residents’ noise exposure under different activities. Noise exposure was high during the shopping, the travel, the social, work or business, exceeding 50 dB on workday and weekend. While noise exposure during sleep was low, with less than 45 dB on both days. In addition, the noise exposure in personal affairs, family affairs and recreational activities was about 48–49 dB, respectively. The noise exposure was roughly similar under different activity types on workdays and weekends, and varied greatly only during shopping, other activities and social activities; the noise exposure on weekends was higher than that on workdays. Residents’ noise exposure was different under different activity types; the noise tolerance values (A dose that causes discomfort) of residents may also be different under different activity types. Further research on the relationship between noise and mental health should be conducted in specific activity scenarios.

#### 3.3.3. Characteristics of Residents’ Noise Exposure under Different Activity Places

**Figure 8:**
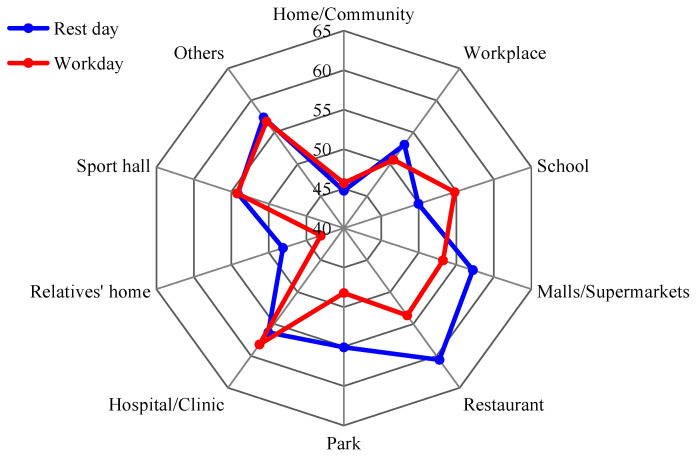
Noise exposure under different activity places on workday and weekend.

Figure 8 showed the equivalent sound level of residents’ noise exposure in different activity places on workday and weekend. Residents in shopping malls, supermarkets, restaurants, hospitals and other activity places were exposed to high noise which exceeded 50 dB on workdays and weekends. On the other hand, the noise exposure was low at home and at relatives’ homes, both between 45 and 50 dB. In addition, the residents’ noise exposure in schools and parks were about 48–55 dB. Compared with the residents’ noise exposure in different activity places on workdays and weekends, the noise exposure was roughly the same for two days only in sports halls, hospitals, clinics, homes, workplaces and other activity locations. Noise exposure was significantly higher on weekends than on workdays in relatives’ homes, parks, restaurants, shopping malls and supermarkets. This may be related to the flow of people and bustle of different activity places on workdays and weekends. Similarly, further research on the relationship between noise and mental health should be conducted in a specific activity space.

### 3.4. Self-Reported Mental Health Characteristics of Residents

**Figure 9:**
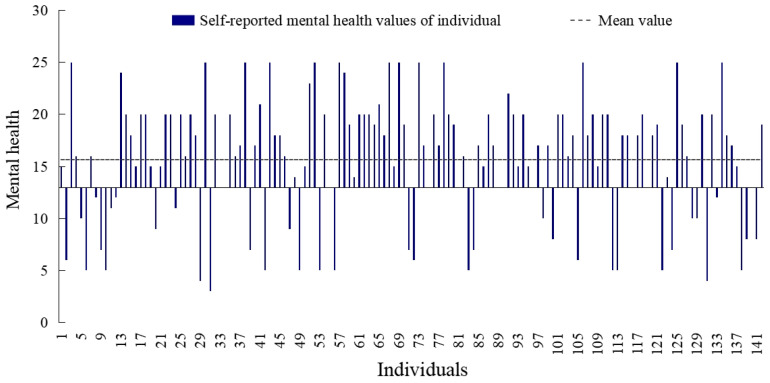
Self-reported mental health values of individual.

Figure 9 showed that the mean value of residents’ self-reported mental health was 15.6. According to the World Health Organization’s Five Well-Being Indexes (WHO-5) [32], a score of less than 13 indicates the person’s mental status is poor. In total, 25.4% of the residents had psychological problems, and their self-rated mental health value was lower than 13.

### 3.5. The Mental Health Effect Thresholds of Spatiotemporal Environmental Noise Exposure

We further analyzed the relationship between noise exposure and residents’ mental health at different times, activities and places by random forest model. It should be noted that the sample size of residents during shopping, social and other activities was too small to be analyzed. Similarly, the relationship between noise exposure and mental health in different activity places was analyzed only at home and in the workplace. Individual attribute variables were taken as control variables in all models, and the relative contribution of noise exposure to mental health is shown in Table 3.

Residents’ noise exposure during sleep (*L_eaq,Sl_*, Model 11) was an important independent variable for mental health, with a 6.06% contribution. Noise exposure at workspace (*L_eaq,WS_*, Model 13), noise exposure during work (*L_eaq,Work_*, Model 6), noise exposure at home or in community (*L_eaq,Home_*, Model 12), noise exposure at travel (*L_eaq,Tra_*, Model 10), noise exposure in personal affairs (*L_eaq,PA_*, Model 7), and noise exposure at night (*L_eaq,N_*, Model 3) had 4.42%, 3.86%, 3.63%, 3.54%, 3.08% and 1.20% contribution towards mental health, respectively. However, the noise exposure over two days (*L_eaq,48h_*, Model 1), noise exposure during daytime (*L_eaq,D_*, Model 2), noise exposure on workdays and weekends (*L_eaq,W_*, Model 4 and L_eaq,R_, Model 5), noise exposure during family affairs (*L_eaq,FA_*, Model 8) and recreational activities (*L_eaq,Re_*, Model 9) had no significant effect on mental health, and their relative importance was negative.

**Figure 10:**
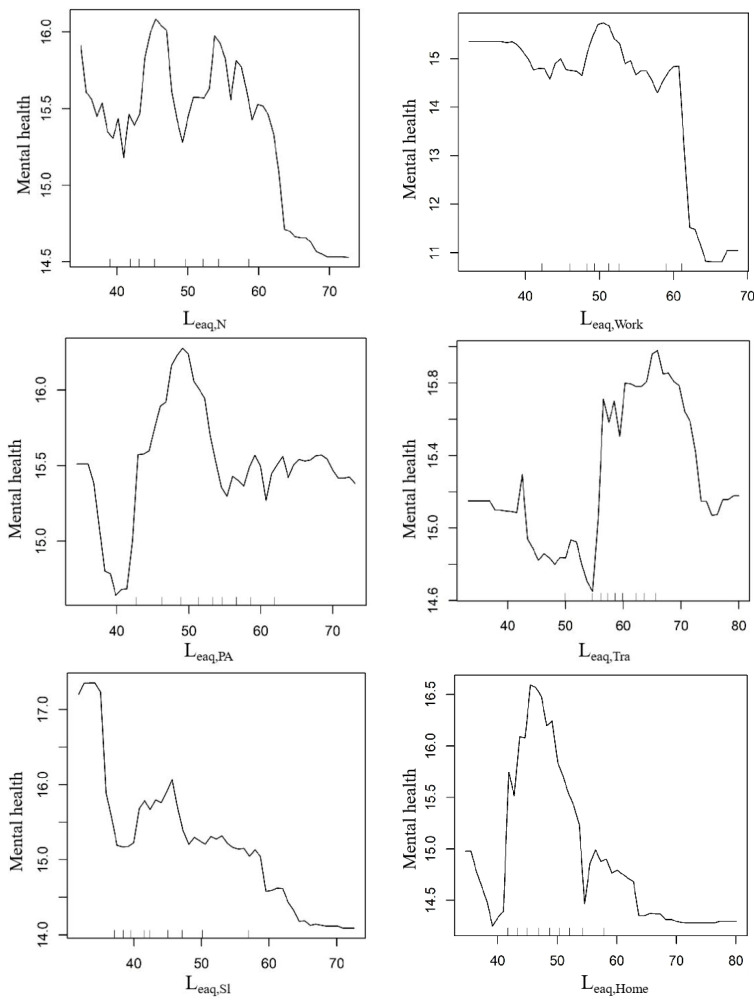

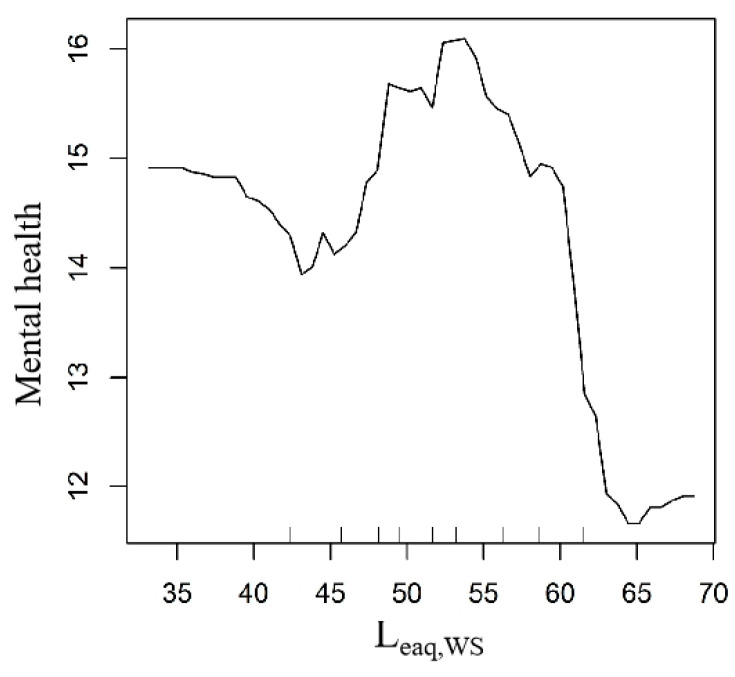
The nonlinear relationship between noise exposure and mental health.

Figure 10 showed the partial dependence between seven noise exposure variables and mental health. The partial dependence plot gives a graphical description of the marginal effect of the variable on the response variable after taking into account the average effect of all other variables in the model.

The difference in self-reported mental health among residents exposed to different levels of noise at night was 1.5 points. When the noise value at night was between 35 and 60 dB, its impact on the mental health level of residents is not clear. In this range, the mental health level fluctuated irregularly with the change of the noise value of about 0.5 points. When the noise value reached about 60 dB, the mental health level decreased significantly, and then decreased slowly until it remained at the lowest level.

The threshold for mental health effects of noise exposure at work was 60 dB. When the noise value was lower than 60 dB, its impact on mental health was very small, and the corresponding mental health value was about 14.5 to 15.5 points. When the noise level reached 60 dB, the mental health level dropped sharply, from 15 points to 11 points. According to the WHO (Five) Well-Being Index (1998 version), a score below 13 indicates poor health. We concluded that the tolerance threshold of noise at work was 60 dB; exceeding this level will lead to mental health problems in residents.

For the three models of noise exposure during personal business, noise exposure during travel, and noise exposure at home, it does not seem that the lower the noise level, the better the mental health. Considering the changes in mental health levels, the optimal sound range was found for personal affairs, travel and staying at home. Among them, when the noise value of personal affairs was around 40 dB, the mental health level was the lowest. However, when the noise value was in the range of 42–50 dB, the mental health level increased with the increase in the noise value. However, when the noise value exceeded 52 dB, the mental health level dropped sharply. When the noise value reached 55 dB, the mental health level dropped to about 15.5 and then stayed basically unchanged. It can be seen that around 50 dB (45–52 dB) seems to be the most comfortable sound level for personal affairs. Similarly, when the noise value was 43–53 dB, the mental health level was at the lowest level. When the noise value was lower than 43 dB or higher than 70 dB, the mental health level was at the middle level. Thus, the noise value of 55–70 dB was the best range of mental health level during travel. The lowest level of mental health was found at home with noise levels lower than 42 dB or higher than 52 dB, whereas the optimal range for mental health was 44–52 dB.

The mental health of residents was most sensitive to noise during sleep, and the difference in the mental health value was 3.4 points. When the noise level increased from 34 dB to 37 dB, the mental health score dropped sharply by about 2.2 points. Subsequently, the mental health score gradually decreased with the increase in the noise value, until the noise value reached 64 dB, the mental health score also fell to the lowest level and basically did not change.

Similar to the model of noise exposure at work, the mental health score dropped significantly from 15 (good health) to 12 (poor health) when noise exposure levels exceeded 60 dB at the workplace. The difference was that when the noise level was between 32 dB and 45 dB, the mental health score decreased slightly with the increase in the noise level. In the noise range of 48–52 dB, the mental health level was the best.

## 4. Discussion

### 4.1. Comparison of the Research Findings with Current Environmental Noise Standards

In this study, the noise threshold identified were 60 dB at night, 60 dB at work, and about 34 dB during sleep, about 50 dB for personal business, 55–70 dB for travel, and 45 dB at home. The optimal sound environment for personal affairs, travel and at home were about 50 dB, 55–70 dB and 45 dB, respectively. Compared with the existing standards for environmental noise, the WHO recommend that the average level of road traffic noise, rail traffic noise and air noise at night should be controlled below 45 dB, 44 dB and 40 dB, respectively [41]. Additionally, according to the Acoustic Environmental Quality Standards (GB 3096-2008), the nighttime noise limits of class 0 acoustic environment functional area (referring to areas that require special quiet such as convalescent area) and class 1 acoustic environment functional area (referring to the areas whose main functions are residential, medical and health care, culture and education, scientific research and design, administrative office, and need to keep quiet) were 40 dB and 45 dB, respectively [42]. The nighttime noise threshold of 60 dB obtained in this study was much higher than the WHO recommendations and the existing nighttime noise standards in China, which may be related to the failure to distinguish noise sources in this study. However, the sleeping noise threshold identified in this study was 45 dB which lower than the WHO recommended nighttime exposure level. It indicated that the key period of noise control at night is during residents’ sleep period, whereas residents may have a higher tolerance to noise derived from activities related to life and entertainment (such as cooking, electrical appliances, talking, TV, mobile phones) during non-sleep period. For example, previous studies showed that the association between road traffic noise and the probability of a high level of sleep disturbances was OR: 2.13 (95% CI: 1.82–2.48) per 10 dB increase in noise [43], but when the noise source was not specified, the probability was OR: 1.09 (95% CI: 0.94–1.27) per 10 dB increase and was no longer statistically significant [44,45,46,47]. It can be verified that lower sound levels are not always better at home and in personal affairs, and the optimal ambient sound values for both were around 45 dB and 50 dB, respectively. According to the noise threshold value of 60 dB during the working period or in the working place, it was higher than the daytime limit value of 55 dB given in the Sound Environment Quality Standard.

Traffic noise has recognized as the main noise source in urban environment, and has been widely proved to have adverse effects on residents’ health [48,49,50,51]. In this study, the optimal sound environment for travel was determined to be 55–70 dB, which was well below the WHO guideline for protecting human hearing (70 dB) (World Health Organization, 1980 [28]) and the day-time limit standard 70 dB for class 4a functional areas (expressways, primary highways, secondary highways, urban expressways, urban trunk roads, urban secondary trunk roads, urban rail transit (surface section), and areas on both sides of inland waterways) in Sound Environment Quality Standards. Some studies have pointed out that the noise threshold of bus passengers’ instantaneous emotional influence is 65–79 dB, and suggested that the noise level should be controlled at 65 dB [31]. It can be found that residents tend to be exposed to a high noise environment when traveling (average value is 55.5 dB), which may lead to residents gradually adapting to a high noise level in the process of traveling, and only when the noise value exceeds the maximum to bring physiological (hearing) impact on them will become sensitive.

### 4.2. Policy Implications

In order to further promote the prevention and control of urban noise pollution and improve residents’ adverse health problems caused by noise, it is necessary to put forward more accurate noise prevention and control measures for urban areas, and encourage residents to take more active noise prevention and control measures. Therefore, the following measures were outlined in this paper as policy basis for urban management departments, ecological environment departments and reference for individual noise prevention and control. More research on noise and health effects is required to develop in the future to form a more reliable policy support and basis.

First of all, it is necessary to strictly control noise levels in key places (such as residential areas) and sensitive time periods (such as while sleeping at night). Soundproof windows and soundproof doors can be installed in residential buildings and office buildings where noise sources are close to each other in living and working areas.

Secondly, differentiated noise control standards should be formulated for different places, and an early warning mechanism should be adopted for important public places with environmental noise. For shopping malls, supermarkets, restaurants and other places, the noise impact threshold of residents can be combined to provide a more comfortable and pleasant reference standard for environmental noise. In stations, squares, buses and other places with high human flow density, especially in important occasions where people gather for major holidays, the early warning mechanism for people with environmental noise should be developed according to the residents’ emotional response to environmental noise exposure.

Finally, it is necessary to strengthen the education around noise hazards and self-prevention and control for residents. There are some differences in noise sensitivity and health hazard awareness among residents. Government management departments should further use social media, neighborhood committees and other media and means to guide residents to avoid and protect themselves against high noise risk, so as to reduce the effect of urban noise on residents’ health.

### 4.3. Strengths and Limitations

This study pays more attention to the threshold effects of environmental noise exposure in their daily activities on mental health. Our research findings highlight the difference in noise threshold in different time, space and activity backgrounds. The result can provide theoretical reference for the formulation of differentiated noise control measures in specific activity places, as well as resident active noise prevention and control and noise health risk intervention.

However, this research still has several limitations. For example, in the threshold study of noise from residents’ daily activities on mental health, the relationship between noise and mental health is complex, which is affected by acoustic and non-acoustic factors. In this study, only individual attributes were considered but some acoustic factors (such as type of noise source and noise frequency) were not considered, which may lead to some changes in the threshold. Secondly, the study only analyzed the threshold relationship between objective environmental noise exposure and mental health. In the future, the objective noise exposure and subjective noise perception of individuals can be further combined for analysis. Thirdly, it ignores the cumulative effects of environmental noise exposures on residents’ mental health over prolonged periods. In the future, the noise threshold on mental health can be comprehensively evaluated by combining the effect of exposure time. Fourthly, home and community have been considered to be the same type in the activity location type. However, noise levels between activities at home and outdoor in the community may be pretty different, and may have differentiated mental health effects.

Finally, limited by the difficulty of data collection, high cost and long-time consumption, this paper only had more than 100 samples in the dynamic environmental exposure monitoring and investigation of residents’ daily activities. It failed to cover more activity types and activity places in the analysis of noise exposure in daily activities and mental health. In future studies, targeted noise threshold research can be conducted according to different activity types, places and times.

## 5. Conclusions

This paper mainly studied the threshold effect of environmental noise exposure on residents’ mental health in their daily activities. The results showed that the noise exposure of residents under daily activities has obvious differences in time, space and place. Noise exposure at night, work, during personal affairs, travel and sleep activities, as well as at home and at the workplace, had a threshold effect on residents’ mental health. Noise thresholds were 60 dB, 60 dB, and about 34 dB at night, during work or at work, and while sleeping, respectively. The optimal sound environment for personal business, travel, and at home were around 50 dB, 55–70 dB, and 45 dB, respectively. The noise thresholds for each time, activity and place determined in this study were different from the existing recommendations and standards. More research is needed to provide a basis for more accurate noise control standards. This study makes an important contribution to explore the noise threshold and optimal acoustic environment level for different times, activities and places. It can also be a policy basis for urban management departments, ecological and environmental departments, and a reference for individual noise prevention and control.

## Figures and Tables

**Table 1:** Description of variables.

Variables	Variable Description	Value Set	Sample Size
Demographic characteristics			
Gender	Male (yes = 1, otherwise = 0)	Categorical variable: {0, 1}	142
Age	Age	Continuous variable: values > 18	142
Education	Highest completed education (senior high school or lower = 0, otherwise = 1)	Categorical variable: {0, 1}	142
Personal income	Personal average monthly income	Continuous variable	142
Physical health	Self-reported physical health (terrible = 1, not good = 2, general = 3, relatively good = 4, health = 5)	Categorical variable: {1, 2, …, 5}	142
Per capita living space	The housing area divided by the number of people living in the house (m^2^)	Continuous variable	142
Greening exposed	Weekly time spent in a green space (park, small garden, community green space, public green belt, etc.)	Continuous variable	142
Neighborhood	Assessed using five questions: “Be familiar with my neighbors?” “I think my neighbors are similar to my ideas?” “I think the neighbors in the community trust each other?” “In trouble, I can find my neighbors to help?” “I think the neighborhood is very harmonious?” Five response categories on a 5-point scale were used: “not at all”, “slightly”, “moderately”, “considerably”, “very much”, with values of 1–5, respectively	Continuous variable: values of 5–25	142
Noise exposure			
*L_eaq,48h_*	A-weighted equivalent sound level values calculated by residents noise exposure on one workday and one weekend day	Continuous variable	142
*L_eaq,D_*	A-weighted equivalent sound level values calculated by residents noise exposure from 6:00 to 22:00 on one workday and one weekend day	Continuous variable	142
*L_eaq,N_*	A-weighted equivalent sound level values calculated by residents noise exposure from 22:00 to 6:00 on one workday and one weekend day	Continuous variable	142
*L_eaq,W_*	A-weighted equivalent sound level values calculated by residents noise exposure on one workday	Continuous variable	142
*L_eaq,R_*	A-weighted equivalent sound level values calculated by residents noise exposure on one weekend day	Continuous variable	142
*L_eaq,Work_*	Equivalent sound level calculated by residents noise exposure at work on one workday and one weekend day	Continuous variable	94
*L_eaq,PA_*	A-weighted equivalent sound level calculated by residents noise exposure during personal business on one workday and one weekend day	Continuous variable	142
*L_eaq,FA_*	A-weighted equivalent sound level calculated by residents noise exposure during family affairs on one workday and one weekend day	Continuous variable	124
*L_eaq,Shop_*	A-weighted equivalent sound level calculated by residents noise exposure during shopping on one workday and one weekend day	Continuous variable	43
*L_eaq,Re_*	A-weighted equivalent sound level calculated by residents noise exposure during recreational activities on one workday and one weekend day	Continuous variable	119
*L_eaq,So_*	A-weighted equivalent sound level calculated by residents noise exposure during social activities on one workday and one weekend day	Continuous variable	38
*L_eaq,Tra_*	A-weighted equivalent sound level calculated by residents noise exposure during travel on one workday and one weekend day	Continuous variable	136
*L_eaq,Sl_*	A-weighted equivalent sound level calculated by residents noise exposure during sleep on one workday and one weekend day	Continuous variable	142
*L_eaq,OA_*	A-weighted equivalent sound level calculated by residents noise exposure during other activities on one workday and one weekend day	Continuous variable	20
*L_eaq,Home_*	A-weighted equivalent sound level calculated by residents noise exposure at home or community on one workday and one weekend day	Continuous variable	142
*L_eaq,WS_*	A-weighted equivalent sound level calculated by residents noise exposure at workplace on one workday and one weekend day	Continuous variable	87
*L_eaq,Sc_*	A-weighted equivalent sound level calculated by residents noise exposure at school on one workday and one weekend day	Continuous variable	28
*L_eaq,Ma_*	A-weighted equivalent sound level calculated by residents noise exposure at the mall on one workday and one weekend day	Continuous variable	55
*L_eaq,Res_*	A-weighted equivalent sound level calculated by residents noise exposure at the restaurant on one workday and one weekend day	Continuous variable	48
*L_eaq,Park_*	A-weighted equivalent sound level calculated by residents noise exposure at the park on one workday and one weekend day	Continuous variable	32
*L_eaq,Hos_*	A-weighted equivalent sound level calculated by residents noise exposure at the hospital on one workday and one weekend day	Continuous variable	5
*L_eaq,RH_*	A-weighted equivalent sound level calculated by residents noise exposure at relatives home on one workday and one weekend day	Continuous variable	16
*L_eaq,SH_*	A-weighted equivalent sound level calculated by residents noise exposure at the sports field on one workday and one weekend day	Continuous variable	10
*L_eaq,OS_*	A-weighted equivalent sound level calculated by residents noise exposure at other space on one workday and one weekend day	Continuous variable	58
Dependent variable			
Mental health	WHO-5 based on 5 questions about feeling of “cheerful and in good spirits”, “calm and relaxed”, “active and vigorous”, “fresh and rested”, “daily life has been filled with things that interest me” during the past half of Month, with scale from 0 (none of the time) to 5 (all of the time)	Continuous variable: values of 0–25	142

**Table 2:** The relationship between activity type and the time spent of residents on workdays and weekends.

Activity Types	Workdays		Weekends	
	The Time Spent (h)	Proportion (%)	The Time Spent (h)	Proportion (%)
Work or business	4.53	18.87	1.52	6.32
Personal affairs	12.29	51.22	12.85	53.54
Family affairs	2.50	10.42	3.39	14.11
Shop	0.12	0.49	0.30	1.24
Recreation	2.44	10.19	3.60	15.02
Social	0.31	1.27	0.61	2.55
Others	0.17	0.70	0.22	0.91
Travel	1.64	6.84	1.52	6.32

**Table 3:** Relative contributions of noise exposure variables on mental health.

Model	Variables	Sample Size	Rank	Relative Importance (%)
Model 1	L_eaq,48h_	142	8	-
Model 2	L_eaq,D_	142	8	-
Model 3	L_eaq,N_	142	7	1.20
Model 4	L_eaq,W_	142	8	-
Model 5	L_eaq,R_	142	8	-
Model 6	L_eaq,Work_	94	3	3.86
Model 7	L_eaq,PA_	142	6	3.08
Model 8	L_eaq,FA_	124	8	-
Model 9	L_eaq,Re_	119	8	-
Model 10	L_eaq,Tra_	136	5	3.54
Model 11	L_eaq,Sl_	142	5	6.06
Model 12	L_eaq,Home_	142	6	3.63
Model 13	L_eaq,WS_	87	3	4.42

## Data Availability

The data are not publicly available due to privacy.
